# Experimental Dengue Virus Type 4 Infection Increases the Expression of MicroRNAs-15/16, Triggering a Caspase-Induced Apoptosis Pathway

**DOI:** 10.3390/cimb45060291

**Published:** 2023-05-26

**Authors:** Samir Mansour Moraes Casseb, Karla Fabiane Lopes de Melo, Carlos Alberto Marques de Carvalho, Carolina Ramos dos Santos, Edna Cristina Santos Franco, Pedro Fernando da Costa Vasconcelos

**Affiliations:** 1 Experimental Pathology Section, Evandro Chagas Institute, Ananindeua 67030-000, PA, Brazil; 2 Oncology Research Center, Federal University of Pará, Belém 66075-110, PA, Brazil; 3 Department of Pathology, University of Pará State, Belém 66050-540, PA, Brazil

**Keywords:** apoptosis, dengue virus (DENV), microRNAs, viral nonstructural proteins

## Abstract

The World Health Organization has estimated the annual occurrence of approximately 392 million dengue virus (DENV) infections in more than 100 countries where the virus is endemic, which represents a serious threat to humanity. DENV is a serologic group with four distinct serotypes (DENV-1, DENV-2, DENV-3, and DENV-4) belonging to the genus *Flavivirus*, in the family *Flaviviridae*. Dengue is the most widespread mosquito-borne disease in the world. The ~10.7 kb DENV genome encodes three structural proteins (capsid (C), pre-membrane (prM), and envelope (E)) and seven non-structural (NS) proteins (NS1, NS2A, NS2B, NS3, NS4A, NS4B, and NS5). The NS1 protein is a membrane-associated dimer and a secreted, lipid-associated hexamer. Dimeric NS1 is found on membranes both in cellular compartments and cell surfaces. Secreted NS1 (sNS1) is often present in patient serum at very high levels, which correlates with severe dengue symptoms. This study was conducted to discover how the NS1 protein, microRNAs-15/16 (miRNAs-15/16), and apoptosis are related during DENV-4 infection in human liver cell lines. Huh 7.5 and HepG2 cells were infected with DENV-4, and miRNAs-15/16, viral load, NS1 protein, and caspases-3/7 were quantified after different durations of infection. This study demonstrated that miRNAs-15/16 were overexpressed during the infection of HepG2 and Huh 7.5 cells with DENV-4 and had a relationship with NS1 protein expression, viral load, and the activity of caspases-3/7, thus making these miRNAs potential injury markers during DENV infection in human hepatocytes.

## 1. Introduction

The World Health Organization (WHO) states that about 392 million dengue virus (DENV) infections occur every year in more than 100 countries where the virus is common, representing a severe threat to public health. DENV causes the most common mosquito-borne disease in the world. It consists of a group of four serotypes (DENV-1, DENV-2, DENV-3, and DENV-4) that belong to the genus *Flavivirus*, in the family *Flaviviridae*. Dengue fever (DF) and severe dengue (SD) (formerly called dengue hemorrhagic fever/dengue shock syndrome (DHF/DSS)) are major causes of illness in tropical and subtropical areas [1,2,3].

DENV is spread by the *Aedes aegypti* mosquito, which must feed on a person with the virus during the first 5 days of high viremia, when the person is just starting to feel sick. Asymptomatic individuals can still infect mosquitoes, and infected mosquitoes incubate the virus. The virus remains in the mosquitoes for 8–12 extra days (extrinsic incubation period) before it can be passed on to a susceptible human. The mosquito is infected for its whole life and, more importantly, can spread DENV to humans more than once. There is a lack of evidence as to whether the virus can be passed on in unusual ways, such as through organ transplants, blood transfusions, or from an infected pregnant woman to her unborn child [4].

DENV has a genome of about ~10.7 kb that encodes a single polyprotein, which is post-translationally cleaved into three structure proteins (capsid (C), pre-membrane (prM), and envelope (E)) and seven non-structural (NS) proteins (NS1, NS2A, NS2B, NS3, NS4A, NS4B, and NS5). These take the form of icosahedral, lipid-wrapped particles with a size of 50 nm and the same structure and pathogenic properties but different genetic and serological properties [5].

The NS1 protein is a membrane-associated dimer and a secreted, lipid-associated hexamer (sNS1). Dimeric NS1 is found on membranes in cellular compartments and on the cell surfaces of virus-infected cells. It has been shown that serum from patients contains sNS1, and the levels of this protein are often very high, which is linked to the start of SD. NS1 also affects the innate immune response and has been seen in multiple interactions with the complement system through factor H [6,7,8].

Identifying regulatory ~22 nucleotide (nt) non-coding RNAs is a significant molecular biology task. MicroRNAs (miRNAs) are produced from transcripts that form a fold-back hairpin structure, which is cropped by the RNase III enzyme Drosha to a ~70 nt long pre-miRNA [9,10,11].

This structure is exported to the cytoplasm and further processed by a second RNase III enzyme, Dicer, into the mature ~22 nt miRNA and its complementary star sequence. Mature miRNAs control gene expression by directing effector complexes of Argonaut (Ago) proteins to their cognate targets, which stops translation or speeds up mRNA degradation [12]. Regulation depends on base pairing between the miRNA and target sites on the mRNA. The seed sequence, nucleotides 2–7 at the 5’ end of the miRNA, is significant for this interaction [10,13].

To develop effective diagnostic and therapeutic measures, it is important to better understand the mechanisms of viral replication. In this sense, it is uncertain whether cellular miRNAs are involved directly (acting on the viral genome itself) or indirectly (acting on cellular mRNAs related to processing this protein).

Studies such as that of Jayathilaka et al. [14] suggest that patients with more severe forms of dengue have different NS1 and anti-NS1 rates, which may contribute to the severity of the disease and thus have implications for treatment, vaccination, and diagnostic approaches.

During DENV infection, it is possible to verify the multifactorial role of NS1, including immune evasion, viral replication, and severe dengue pathogenesis [15]. Despite the studies that have been conducted, several important questions remain unanswered, including the molecular determinants of pathogenesis, NS1’s relationship with cellular components, and the action of NS1 in its various functions.

To illustrate the need for studies on mechanisms involving miRNA and NS1 during DENV infection, one can cite the study of Lee et al. [16], who found that the positive expression of Let-7a could be related to the NS1 region of DENV-2 and could inhibit DENV-2 replication.

In this study, we demonstrated a correlation between the expression profile of the NS1 protein and the expression of miRNAs-15/16 during infection with DENV-4 in the human liver cell lines HepG2 and Huh 7.5.

## 2. Materials and Methods

### 2.1. Cell Culture

HepG2 and Huh 7.5 hepatic human cell lines were cultured at 37 °C in a nutrient Dulbecco’s modified Eagle’s medium (DMEM) (Sigma-Aldrich, Burlington, MA, USA) containing L-glutamine, HEPES buffer, and 10% fetal bovine serum (FBS, Gibco, New York, NY, USA).

### 2.2. Viral Samples

At first, the DENV-4 (BeH778494) isolate was grown in C6/36 cells with 5% FBS in Leibovitz 15 (L-15) medium at 28 °C. On the seventh day after infection, the culture supernatant was harvested and stored at −80 °C before the infection of Huh 7.5 and HepG2 cell types.

### 2.3. Infection of Hepatic Human Cells

The HepG2 and Huh 7.5 cells were infected with DENV-4 using the adsorption method for one hour at 37 °C (MOI = 1). Subsequently, the cell cultures were washed with phosphate-buffered saline (PBS), placed into a new medium, and grown again for the experiment. The extracted RNAs were kept at −80 °C until needed. The supernatants of the virus-infected cells were collected daily up to 120 h post-infection (hpi).

### 2.4. RNA Extraction

The cell supernatant was subjected to viral RNA extraction for viral load quantification using the commercial Maxwell 16 Viral Total Nucleic Acid Purification kit (Promega, Madison, WI, USA). The samples were then quantified using the commercial Qubit RNA High Sensitivity (HS) kit (Thermofisher, Waltham, MA, USA) on the Qubit 3.0 platform (Thermofisher, Waltham, MA, USA). The extracted RNAs were kept at −80 °C until needed.

### 2.5. Quantification of Viral Load by RT-qPCR

The cell supernatant was used in each of the analyzed periods. The detection of the DENV genome was performed by RT-qPCR using the method described by Johnson et al. [17] with the commercial GoTaq Probe 1-Step RT-qPCR System (Promega, Madison, WI, USA) on the ViiA 7 platform (Life Technologies, Carlsbad, CA, USA). To quantify the viral load, a standard curve based on the DENV genome previously cloned in the pGEM Easy plasmid (Promega, Madison, WI, USA) was used.

### 2.6. Quantification of miRNA Levels by RT-qPCR

The commercial TaqMan MicroRNA Cells-to-CT kit (Ambion, Carlsbad, CA, USA) was used to measure the levels of miRNAs-15/16, according to the manufacturer’s instructions. Endogenous controls were created from the targets of RNU48 (SNORD48) and RNU58a (the gene for ribosomal protein L17). Quantitative PCR was performed on the ViiA 7 platform (Life Technologies, Carlsbad, CA, USA).

Relative quantification was performed using the method described by Livak et al. [18]. To visualize the miRNA quantification data, Expression Suite v1.0 (Applied Biosystems, Carlsbad, CA, USA) and the R Project’s qPCR.Ct package were used. Each of the experimental durations analyzed was performed in biological triplicate to ensure the reliability of the data.

### 2.7. NS1 Quantification

The amount of NS1 protein was measured with the commercial Platelia Dengue NS1 Ag kit (Bio-Rad, Hercules, CA, USA) and a protein curve built with a synthetic protein made from an expression plasmid. This quantification was performed using supernatants from cells infected with DENV-4.

### 2.8. Apoptosis Detection

To quantify the activity of caspases-3/7, a plate assay was performed with DENV-infected cells and the commercial Caspase-Glo 3/7 Assay kit (Promega, Madison, WI, USA) on the Glomax-Multi+ platform (Promega, Madison, WI, USA), according to the manufacturer’s instructions. The values of the uninfected control (MOCK) were compared with the infection times. This experiment was carried out in technical and biological triplicate.

### 2.9. NS1 Fluorescent Imaging

Up to 120 h after DENV-4 infection, Huh 7.5 cells were moved to a microscope slide and fixed with 3.7% formaldehyde. Subsequently, 3% bovine serum albumin (Sigma-Aldrich) was used to block nonspecific antibody binding sites for 1 h. The cells were placed in a 1:20 solution of FITC-conjugated DENV NS1 polyclonal antibody (Biorbyt, Orwell Furlong, Cambridge, UK) at 4 °C for 16 h. After positioning the coverslip, samples were visualized on an LSM 510 META laser-scanning confocal fluorescence microscope (Carl Zeiss, Colony, Germany) with excitation at 488 nm and emissions collected from 500 to 550 nm. Images were processed using ImageJ v1.48 (National Institutes of Health, Bethesda, MA, USA).

### 2.10. Statistical Analysis

Expression Suite v1.0 (Applied Biosystems, Carlsbad, CA, USA) and the R Project’s qPCR.Ct package were used to statistically analyze the data. Analysis of variance (ANOVA) and Pearson correlation were performed on the Jamovi Project 2.3 platform (jamovi.org accessed on 1 December 2022). *P* values less than 0.05 were considered statistically significant.

## 3. Results

### 3.1. Viral Load Profile during Infection

The viral quantification data were critical to demonstrate both the significant increase during the period of infection and that the two cell lines had qualities for the maintenance of viral replication for the duration of our experiment.

**Figure 1:**
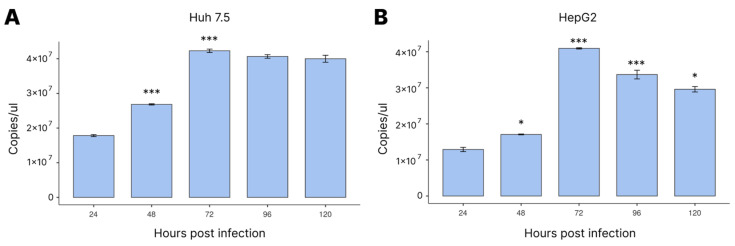
Quantifying viral load in the culture supernatant by RT-qPCR in the ViiA 7 platform as a function of time after DENV-4 infection in Huh 7.5 (**A**) and HepG2 (**B**) cells. Cells were infected with MOI = 1. Statistical analysis was performed using ANOVA with a Tukey post hoc test. * *p* < 0.05, *** *p* < 0.001.

We first looked at the number of virus particles in DENV-4-infected cell lines up to 120 hpi, to find out when the number of viruses increased the most (Figure 1). As observed, 72 hpi was when both cell lines had the highest viral load.

It is noteworthy that the cell lines in the study began to show a difference in viral load at 48 hpi, reaching the highest viral load at 72 hpi, as already reported. However, in the HepG2 strain, it was possible to verify a significant reduction in the viral load from 96 hpi. This study demonstrated a slight variation in viral replication between the two cell lines during the DENV infection.

### 3.2. Expression Levels of miRNAs-15/16 during Infection

Due to its involvement in regulating vital processes, such as growth and development, cell differentiation, and cell apoptosis, miRNA quantification is useful to better understand DENV replication.

Thus, using targets for miRNA-15 and 16, we verified the action of these miRNAs related to apoptosis during DENV infection compared with non-infected controls.

At different time points after DENV-4 infection, qRT-PCR was used to assess the levels of miRNAs-15/16 in the culture supernatants of the Huh 7.5 and HepG2 cells.

**Figure 2:**
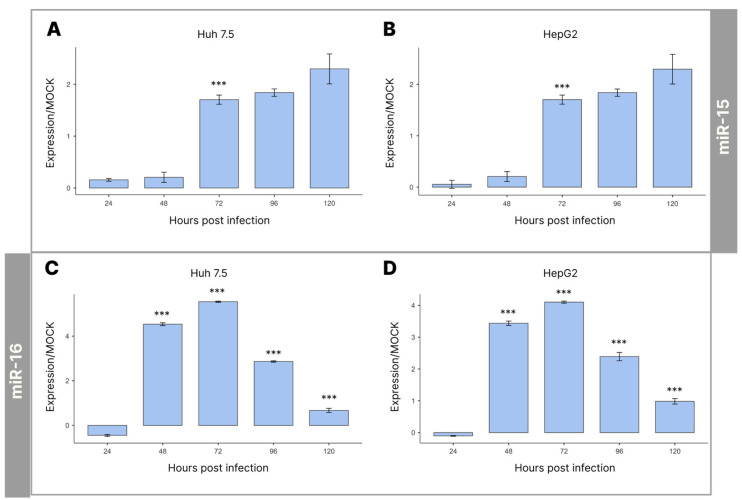
Quantification of miRNA-15 (**A**,**B**) and miRNA-16 (**C**,**D**) levels as a function of time after infection of Huh 7.5 (**A**,**C**) and HepG2 (**B**,**D**) cells with DENV-4 by RT-qPCR with ViiA 7 platform. Cells were infected with MOI = 1. Statistical analysis was performed using ANOVA with a Tukey post hoc test. *** *p* < 0.001.

The expression of miRNA-15 showed a slight change between 24 and 48 hpi. However, the most significant increase was at 72 hpi; after this substantial increase, the miRNA-15 levels remained stable (Figure 2A,B).

In both studied cells, the expression of miRNA-16 showed a significant increase at 48 hpi, which continued until reaching the highest level at 72 hpi. However, there was a significant reduction at 96 hpi, reaching the lowest miRNA-16 level at 120 hpi (Figure 2C,D). Even though both cell lines showed negative expression values for this miRNA at 24 hpi, there was no statistically significant difference between these values and those at the time point before infection, which we called 0 hpi.

The miRNA-15 after 72 hpi presented stable expression, without significant changes after this period, in both cell lines. However, when comparing with the results of miRNA-16, it was possible to verify that after 72 hpi, when the highest expression of this miRNA occurred, a significant reduction began at 96 hpi and continued until the end of the experiment. Thus, similar behavior for miRNA-15 and 16 was possible up to 72 hpi, followed by a change in the expression profile of these miRNAs.

### 3.3. Activation of Caspases-3/7 during Infection

**Figure 3:**
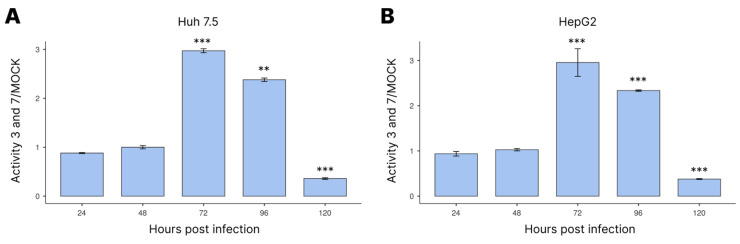
Quantification of caspase-3/7 activity as a function of time after infection of Huh 7.5 (**A**) and HepG2 (**B**) cells with DENV-4 by luminescent assay using the GlomaxMult+ platform. Cells were infected with MOI = 1. Statistical analysis was performed using ANOVA with a Tukey post hoc test. ** *p* < 0.01, *** *p* < 0.001.

Next, we investigated how the apoptosis pathway was turned on during DENV-4 infection by measuring the activity of caspases-3/7 (Figure 3). The results showed that the caspase-3/7 activity was highest at 72 hpi in both cell lines. This activity then declined at 96 hpi and reached its lowest level at 120 hpi.

### 3.4. NS1 Expression during Infection

**Figure 4:**
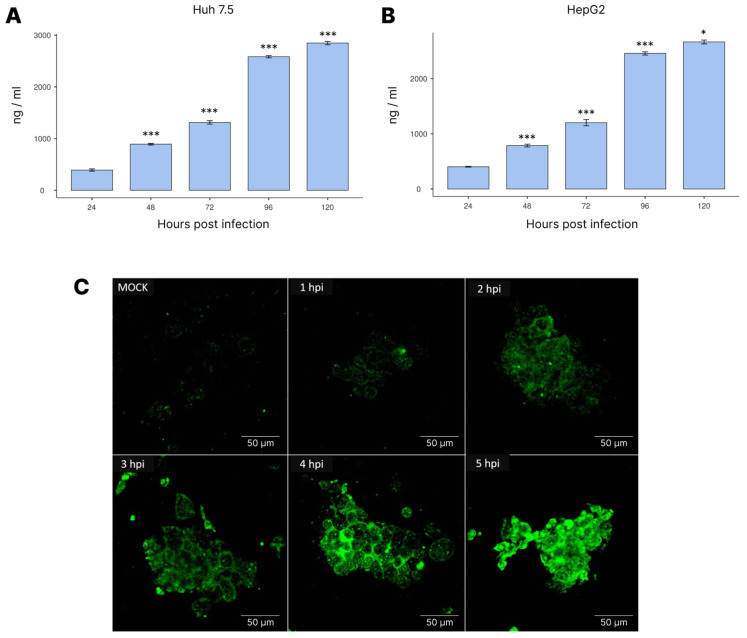
Quantification of NS1 levels in Huh 7.5 (**A**,**C**) and HepG2 (**B**) cells following DENV-4 infection by enzyme immunoassay (**A**,**B**) using the GlomaxMult+ platform; laser-scanning confocal fluorescence microscopy using FITC-conjugated DENV NS1 polyclonal antibody (**C**). Statistical analysis was performed using ANOVA with a Tukey post hoc test. Cells were infected with MOI = 1. * *p* < 0.05, *** *p* < 0.001.

Afterwards, we evaluated the expression of the NS1 protein during DENV-4 infection in HepG2 and Huh 7.5 cells (Figure 4). As observed, the NS1 levels were similar for both cell lines in this experiment, continuously increasing throughout the course of the viral infection.

### 3.5. Correlation among Viral Load, NS1 Levels, miRNA-15/16 Expression, and Caspase-3/7 Activity

We verified that the results obtained in the analysis of viral load, NS1 levels, miRNA-15/16 expression, and caspase-3/7 activity could be related, thus creating an essential profile for the understanding of DENV infection.

**Figure 5:**
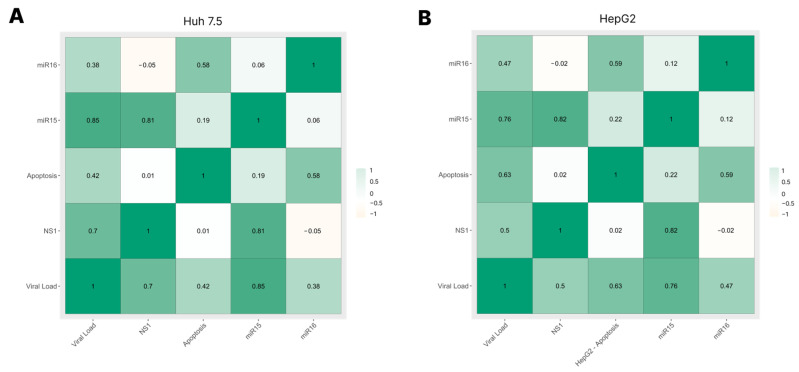
Pearson correlation with Bonferroni methods: analysis between viral load, NS1 levels, miRNA-15/16 expression, and caspase-3/7 activity during DENV-4 infection in Huh 7.5 (**A**) and HepG2 (**B**) cell lines. The values presented in each square are the interaction values between the variables, and the greater the intensity of the green color, the stronger the relationship between the variables.

An analysis of the correlations among the previously obtained data was performed (Figure 5). We observed that the Huh 7.5 and HepG2 cell lines behaved similarly, with a high positive correlation between miRNA-15 expression and both NS1 levels and viral load and a moderate positive correlation between miRNA-16 expression and caspase-3/7 activity.

Our correlation test demonstrated an exciting and promising relationship between viral load, NS1 levels, miRNA-15/16 expression, and apoptosis. The results showed that miRNAs seemed to influence apoptosis during DENV infection. At the same time, we also found that viral load and NS1 levels demonstrated a strong connection with each other and with the apoptosis process, which was quite interesting.

## 4. Discussion

DENV is a *Flavivirus* with a vast worldwide dispersion. Even with the advances in knowledge about this virus, the answers to several questions remain obscure: along with the role of miRNA in DENV infection, one of these questions is whether viral proteins can act as signaling factors.

Studies such as those by Casseb et al. [19] and Castillo and Urcuqui-Inchim [20] showed that DENV infection alters the way in which the main proteins related to miRNA biogenesis are controlled. The differential expression of these proteins during infection with other *Flaviviruses* has previously been observed, as in a study by Holanda et al. [21], who observed a significant change in AGO2 during YFV infection.

Schafer et al. [22] described that members of the miRNA-15/16 family, more specifically miR-424, can suppress the expression of the E3 ubiquitin ligase SIAH1, which is normally induced during DENV-2 infection through the activation of the unfolded protein response (UPR), thus suggesting that miRNAs belonging to the miRNA-15/16 family have an important role during DENV replication.

It should be noted that miRNAs are important in regulatory networks that shape effective T-cell responses by fine-tuning thousands of genes. Studies in mice have demonstrated that miRNAs of the miRNA-15/16 family restrict the cycle, survival, and differentiation of memory T cells [23]. Despite not being the focus of our work, this demonstrates that this family of miRNAs plays an important role in cell cycles and survival.

Interestingly, viral proteins may be related to miRNA [24]. One of these proteins is NS1, which is released from infected cells and can be taken up and stored by hepatocytes. As there is evidence that DENV affects liver function, especially in SD, and NS1 interacts with hepatocytes, these cells are suitable for studying and testing how NS1 is involved in dengue immunopathology. In addition, there is evidence that NS1 can alter cell signaling pathways to help produce more viruses and bypass host defenses [8,25,26].

This study analyzed how miRNA-15/16 expression and NS1 protein levels changed over time during the infection of Huh 7.5 and HepG2 cells with DENV-4. When the virus replicated in these human hepatocyte cell lines, we observed viral load profiles similar to those described by Alhoot et al. [27].

It has been shown that miRNA-15 is a key regulator of apoptosis, and the low expression of this miRNA has been linked to cancer [28,29]. Papillomavirus infections have also been shown to alter the levels of miRNA-15 in cells, having important effects on the p53 signaling pathway [30,31,32]. Even though studies have shown that DENV infection promotes apoptosis [33,34], the molecular basis of this induction is not well-understood.

Like miRNA-15, miRNA-16 is also involved in apoptotic pathways and cell growth during oncogenic processes [23,33,35,36]. The miRNA-16 levels have also been investigated in viral infections. A recent study by El-Abd et al. [37] showed that its regulatory function is diminished when the hepatitis C virus causes a long-term infection, which can lead to hepatocellular carcinoma. In this study, however, we showed that the expression of both miRNAs increased during acute DENV-4 infection, along with an increase in the expression of the NS1 protein and the activity of the apoptosis markers caspases-3/7.

According to [38], miRNAs-15/16 promote the downregulation of Bcl-2—a family of proteins that prevent cell death—by a direct (miRNA–mRNA complementarity) or indirect interaction, which leads to a change in the cell cycle and then apoptosis. In our study, DENV-4 NS1 levels increased along with caspase-3/7 activity up to 72 hpi (Figure 3), suggesting that this viral protein plays a role in apoptosis during DENV-4 infection in liver cells. This finding may be clinically important and may help explain why apoptosis is observed in the liver cells of people who have died from dengue and yellow fever [29,39].

Furthermore, NS1 can interact with Beclin-1 during DENV infection, and this interaction attenuates Beclin-1 cleavage and facilitates autophagy to prevent cell apoptosis [40]. However, as shown by Lu et al. [41], elevated caspases trigger apoptosis by degrading Beclin-1 in the late stage of infection.

Antibodies against NS1 can produce a protective immune response and induce complement-fixing activity. When released into the extracellular medium, NS1 acts as a pathogen-associated molecular pattern (PAMP), which directly activates macrophages and peripheral blood mononuclear cells (PBMCs) via Toll-like receptor 4 (TLR-4), causing them to produce and release pro-inflammatory cytokines and chemokines [26,42].

It was previously shown that, during DENV-1 infection in HepG2 cells, the NS1 protein can be found in lipid raft domains on the host cell surface, suggesting its involvement in signal transduction events [43]. In addition, the authors of this study found that the NF-kB p65 protein moves to the nucleus when NS1 is expressed in HepG2 cells. More DENV infections have recently been linked to NF-kB activation, which leads to endothelial cell death by apoptosis and bleeding symptoms [31,44].

## 5. Conclusions

This study showed that miRNAs-15/16 are overexpressed during DENV-4 infection in human hepatocyte cell lines. NS1 protein production, viral load, and apoptosis were linked to this finding, suggesting a critical interplay between them. Thus, we consider that miRNAs-15/16 have significant potential for use as disease markers during DENV infection in human hepatocytes.

## Data Availability

The data presented in this study are available on request from the corresponding author.
